# Statistical Fragility of Findings From Randomized Phase 3 Trials in Pediatric Oncology

**DOI:** 10.1002/cam4.70356

**Published:** 2024-12-15

**Authors:** Hannah E. Olsen, Pei‐Chi Kao, Caleb Richmond, David S. Shulman, Wendy B. London, Steven G. DuBois

**Affiliations:** ^1^ Department of Pediatrics Boston Children's Hospital and Harvard Medical School Boston Massachusetts USA; ^2^ Dana‐Farber/Boston Children's Cancer and Blood Disorders Center Harvard Medical School Boston Massachusetts USA

## Abstract

**Purpose:**

The fragility index (FI) is an adjunctive metric to facilitate the interpretation of *p*‐values in clinical trials. The FI has not been studied in phase 3 trials in pediatric oncology.

**Methods:**

PubMed was used to identify phase 3 pediatric oncology trials published between 1980 and 2020. We report trial characteristics and calculate the FI for trials with a binary outcome and survival‐inferred fragility index (SIFI) for trials with a time‐to‐event outcome. FI/SIFI is the number of patients from one arm of a trial who would need to change groups for the statistical conclusion to change. We also report fragility quotients (FQ and SFQ) to normalize FI and SIFI relative to trial size.

**Results:**

One hundred and thirteen trials included sufficient data for analysis. The median FI for trials with a binary outcome (*n* = 40) was 4.5 (range: 1–33). The median SIFI for trials with a time‐to‐event outcome (*n* = 73) was 13 (range: 0–61). The FI or SIFI was less than the number of patients lost to follow‐up in 25% of 36 trials. Median FQ and SFQ were 0.026 and 0.03, respectively, and did not significantly vary according to trial characteristics. While sample sizes increased over time, the FQ and SFQ remained stable.

**Conclusions:**

The statistical conclusions of pediatric oncology phase 3 trials hinge on a relatively small number and proportion of patients. Despite the sample size limitations of low prevalence diseases, pediatric cancer trials are similarly or less fragile than adult oncology trials. Smaller trials do not appear more statistically fragile than larger trials. Statistical fragility appears to have remained constant over the four decades evaluated. We recommend reporting FI or SIFI, in conjunction with *p*‐values, for all phase 3 pediatric oncology trials.

## Graphical abstract

On average, 13 patients can change the statistical conclusion of a trial in pediatric oncology. Pediatric oncology trials are similarly or less statistically fragile than adult oncology trials. Statistical fragility has not changed over the last four decades.

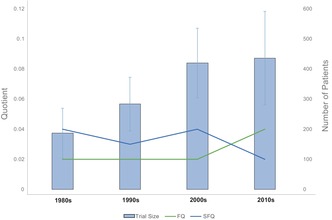

## 1 Introduction

Phase 3 clinical trials are the gold standard for studying therapeutic efficacy and often provide important comparative data used by regulatory authorities for new drug approvals. The relative success of these trials is dependent upon falling below a predetermined statistical probability that observed findings are unlikely to be due to chance alone (typically *p* < 0.05), a practice that has been critiqued for its inability to extrapolate the magnitude of clinical benefit conferred by an intervention [1, 2]. In 2014, the concept of a “fragility index” (FI) was developed by Walsh and colleagues as a means to more comprehensively evaluate the statistical robustness and internal reliability of a study and in turn facilitate a more nuanced interpretation of trial results [3]. Simply put, the FI represents the number of patients on the experimental arm of a positive randomized trial who would need to switch from “responder” to “nonresponder” in order for the comparison to the standard arm to lose statistical significance. Conversely, this calculation can be performed in reverse to determine the number of patients required for a nonsignificant trial to become significant. These methods have since been used to study trials in many fields of medicine including cardiology [4], surgery [5, 6], and pediatrics [7].

More recently, an analysis of randomized phase 3 trials used to support FDA approval of anticancer agents yielded a median FI of 2, and found that roughly half of the trials had an FI representing less than 1% of the sample size [8]. One limitation of this analysis was the failure to accommodate time‐to‐event endpoints (e.g., event‐free or progression‐free survival), commonly used in oncology trials [9, 10]. In response, Bomze and colleagues developed a modified “survival‐inferred fragility index” (SIFI) based on a reverse‐engineering strategy to estimate the number of patients who had an event at an average exposure time, thus accommodating time‐to‐event endpoints [11]. This SIFI methodology has been applied to medical oncology trials, including one report focused on phase 3 trials of immune checkpoint inhibition that found a median SIFI of 5 [12].

To date, there have been no studies of the FI or SIFI specifically in pediatric oncology trials. These metrics are of particular interest in pediatric oncology since trials in this field are inherently limited by smaller enrollment numbers given the decreased prevalence of cancer in children relative to adults [13]. To address this gap, we performed a retrospective analysis of phase 3 pediatric oncology trials conducted over the last four decades to determine FI and SIFI of trials with binary and time‐to‐event outcomes, respectively. We report FI for positive trials and reverse FI for negative trials. Lastly, we report the fragility quotient (FQ), which is the ratio of FI to trial sample size, to better contextualize and standardize FI and SIFI values.

## 2 Methods

### 2.1 Trials and Trial Characteristics

We used PubMed to search the peer‐reviewed literature for potentially eligible randomized phase 3 clinical trials studying a pediatric cancer indication, published from January 1, 1980 to May 1, 2020 (Appendix A).

Trials had to meet the following criteria: randomized controlled trial with experimental arm and comparator arm; known outcome (for trials with time‐to‐event endpoints: event present or absent during the follow‐up period; for trials with binary endpoints: responder or nonresponder); known number of events or responders per arm or Kaplan–Meier curve available to digitize (see below); pediatric or adolescent population (trials summarizing a combined adult/pediatric cohort were only included if the median age of the trial cohort was < 18 years); and patients had a cancer diagnosis (supportive care trials were included only if > 50% of patients had a cancer diagnosis). Trials that did not specify the phase were included if the sample size was > 100 patients (given they met all other criteria), as larger pediatric trials were likely to be phase 3. Trials with a factorial design were excluded. Trials that used precision (the width of a confidence interval) rather than hypothesis testing (a *p*‐value) to address the primary objective were excluded. Trials that met criteria but were missing critical information on statistical design were excluded. Trials with multiple separate primary randomizations in independent patient cohorts were included. For trials with serial randomizations of the same cohort, only the first randomization was included.

Trial characteristics were extracted from the eligible publications. A list of drugs that are FDA approved for a pediatric cancer indication was obtained on May 31, 2022 from https://www.cancer.gov/about‐cancer/treatment/drugs/childhood‐cancer‐fda‐approved‐drugs. The FDA label for each drug was reviewed to determine if any of the randomized trials in this analysis were included in the Clinical Studies section of the label.

### 2.2 Definitions

A positive randomized trial is one which rejects the null hypothesis and is able to conclude the desired result. A negative randomized trial fails to reject the null hypothesis and is unable to conclude the desired result. A randomized superiority trial tests for the benefit of an experimental arm versus a comparator arm, while a randomized noninferiority trial tests for more than an acceptable margin of lesser benefit of an experimental arm versus a comparator arm (Table S1).

### 2.3 Calculation of Fragility Indices (FI)

For superiority trials (experimental arm was superior) and a binary endpoint, we calculated the FI by iteratively moving patients in the experimental (positive) arm from the response category to the nonresponse category until a Fisher's exact test (one‐sided or two‐sided, whichever was used in the original testing) was no longer statistically significant at the *p* < 0.05 level. The number of patients at which statistical significance was lost is the FI. These calculations were performed using the R package *fragilityindex,* which used a Fisher's exact test for comparing the two treatment arms. In trials where the original *p*‐value was nonsignificant (typically *p* ≥ 0.05), a reverse FI was calculated, which is equal to the minimum number of patients required to be reassigned from the nonresponse category to the response category until a significant *p*‐value is obtained. These calculations were performed using R function *revfragility.index*.

### 2.4 Calculation of Survival‐Inferred Fragility Indices (SIFI)

For superiority trials with time‐to‐event endpoints, we reconstructed individual patient data (IPD) by digitizing the published Kaplan–Meier curves; information on the total number of events and number‐at‐risk was obtained using the R Shiny application *IPDfromKM*, per the methods of Liu et al., which applies an assumption of uniform censoring [14, 15]. Then the methods of Bomze et al. were applied to calculate the SIFI using R function *sifi* (if the reconstructed *p*‐value was less than the trial‐specific significance level) or the reverse SIFI using function *neg_sifi* (if the reconstructed *p*‐value was greater than the trial‐specific significance level) [12]. In each case, a log‐rank test (either one‐sided or two‐sided, whichever was used in the trial) was used to compare the two treatment arms (Table S1). A visual representation of this process is shown in Figure S1, an example of a positive superiority trial with SIFI = 6 [16]. The best survivor, defined as the patient with the longest survival time (whether an event or censored) is reassigned from the curve with higher outcome to the curve with lower outcome, resulting in a *p*‐value that gradually increases until it becomes nonsignificant.

For negative noninferiority trials, SIFI is defined as the minimum number of reassignments of the “best survivors” from the experimental group to the control group until the loss of significance. For positive noninferiority trials, a negative SIFI is the minimum number of reassignments of best survivors from the control to the experimental group until the *p*‐value becomes significant.

### 2.5 Data Analysis

Other than the inferential tests for treatment arm comparisons in calculating FI and SIFI, all analyses were descriptive: number, proportion, median, range, and coefficient of determination (*R* ^2^) for simple linear regression. FI, reverse FI, and SIFI values are always positive and do not indicate the direction of reassignment. However, reverse SIFI can take on negative values. To align our ability to interpret and present FI and SIFI, we have presented the absolute value of the reverse SIFI.

We normalized FI and SIFI to adjust for differences in sample sizes across trials by calculating the FQ and survival fragility quotient (SFQ), where FQ and SFQ, respectively, are defined as FI or SIFI divided by the total sample size of the trial. FQ and SFQ reflect the proportion of the sample size that would need to be reclassified to change the original statistical conclusion; therefore, FQ and SFQ range from 0 to 1. Larger values of FQ and SFQ are indicative of more robust results, while smaller FQ and SFQ are indicative of trials with more “fragile” results.

## 3 Results

### 3.1 Trial Characteristics

**FIGURE 1:**
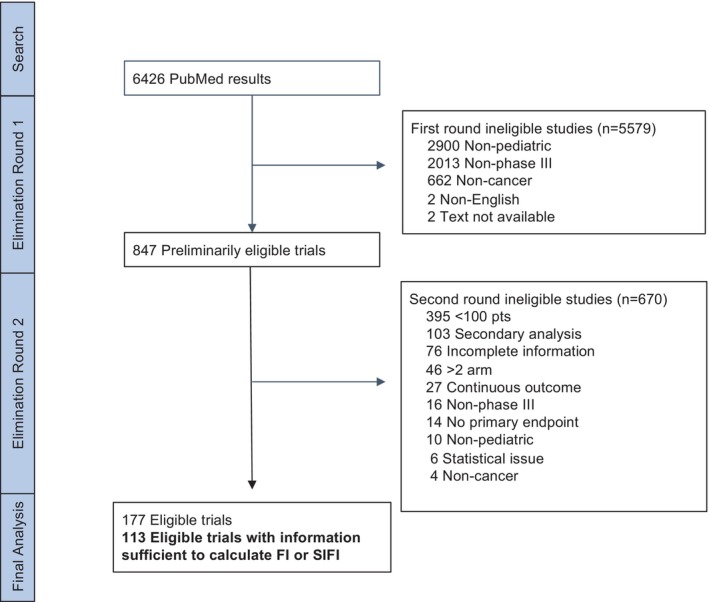
Trial search CONSORT diagram.

A total of 6426 trials were identified in the initial search (Figure 1). After sequential rounds of review, 113 trials met the eligibility criteria (Table 1). Of these 113 trials, the median sample size was 295 patients (range: 53–3720). Mean trial size increased over time from 187 patients in the 1980s to 437 patients in the 2010s. Fifty‐two percent (*n* = 59) of trials focused on a hematologic malignancy indication, 30% (*n* = 34) solid tumor, 5% (*n* = 6) neuro‐oncology, and 12% (*n* = 14) included any cancer type. The majority of trials (78%) studied a direct anti‐cancer intervention; 22% studied a supportive care intervention.

**TABLE 1:** Characteristics of 113 pediatric oncology randomized trials included in fragility index calculations.

	All trials (*n* = 113)	Positive trials (*n* = 41)	Negative trials (*n* = 72)
Type of endpoint—*n* (%)			
Time‐to‐event endpoint	73 (65%)	24 (59%)	49 (68%)
Binary response endpoint	40 (35%)	17 (51%)	23 (32%)
Time‐to‐event data completeness^a^			
KM known, events known, NAR known	40/73 (55%)	12/24 (50%)	28/49 (57%)
KM known, events known, NAR unknown	22/73 (30%)	7/24 (29%)	15/49 (31%)
KM known, events unknown, NAR known	11/73 (15%)	5/24 (21%)	6/49 (12%)
Type of therapy—*n* (%)			
Anticancer therapy	88 (78%)	30 (73%)	58 (80%)
Supportive care	25 (22%)	11 (27%)	14 (20%)
Total sample size of trial–median (range)	295 (53–3720)	293 (53–3720)	313 (54–1941)
Average total sample size of trial—*n*			
1980–1989	187	134	202
1990–1999	284	360	236
2000–2009	420	452	402
2010–2019	437	447	431
Disease category—*n* (%)			
Hematologic malignancy	59 (52%)	20 (49%)	39 (54%)
Solid tumor	34 (30%)	13 (32%)	21 (29%)
Neuro‐oncology	6 (5%)	1 (2%)	5 (7%)
Multiple disease groups	14 (12%)	7 (17%)	7 (10%)
Decade of publication—*n* (%)			
1980–1989	9 (8%)	2 (5%)	7 (10%)
1990–1999	18 (16%)	7 (17%)	11 (15%)
2000–2009	35 (31%)	13 (32%)	22 (31%)
2010–2019	51 (45%)	19 (46%)	32 (44%)
Number of drugs—*n* (%)			
Monotherapy	22 (19%)	9 (22%)	13 (18%)
Combination therapy	80 (71%)	26 (63%)	54 (75%)
Not a trial of a systemic drug	11 (10%)	6 (15%)	5 (7%)
Registration status—*n* (%)			
Used for FDA approval	1 (1%)	1 (2%)	0 (0%)
Not used for FDA approval	112 (99%)	40 (98%)	72 (100%)
Type of trial design			
Superiority design	103 (91%)	35 (85%)	68 (94%)
Noninferiority design	10 (9%)	6 (15%)	4 (6%)

Abbreviations: KM, Kaplan–Meier; NAR, number at risk.

^a^ IFI calculation requires Kaplan–Meier (KM) curve and known data for either total events or number‐at‐risk (NAR). Conventional FI calculation requires known data for both total events and total sample size.

Seventy‐three (65%) trials reported time‐to‐event as the primary endpoint. Forty (35%) trials reported a binary response endpoint. The distribution of trial characteristics was similar between positive and negative trials, except in terms of trial design. Of 72 negative trials, the majority (94%) used a superiority design and only 6% used a noninferiority design, whereas for 41 positive trials, 85% used a superiority design and 15% used noninferiority design. Ten trials were stopped early for futility. Three trials were stopped early for success.

### 3.2 Fragility Index and Quotient for Trials Reporting a Binary Outcome

**FIGURE 2:**
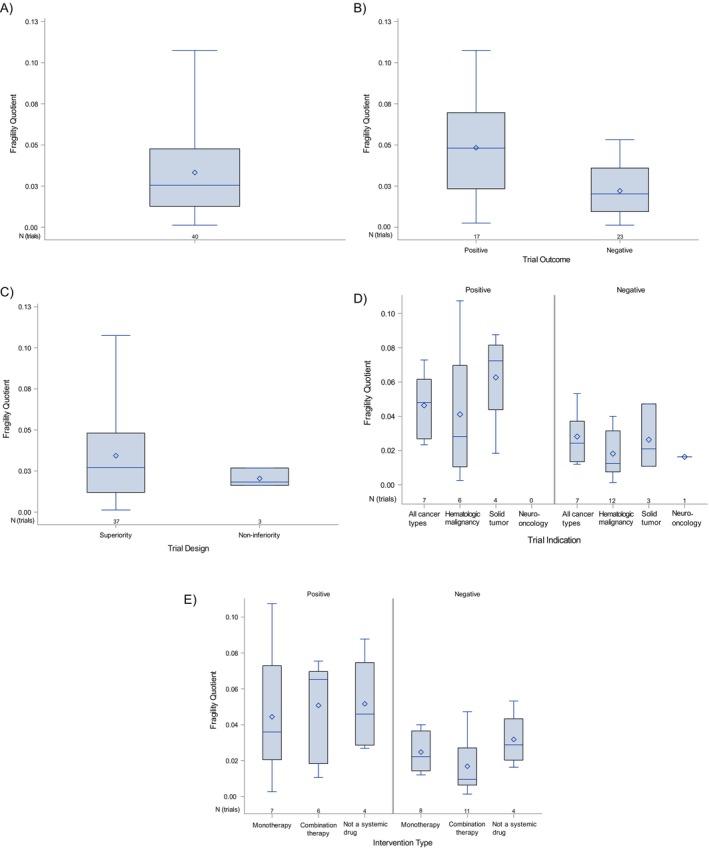
Box and whisker plots, with minimum, maximum, quartiles, and mean of fragility quotients for all trials: With binary endpoints (A); according to trial outcome (B); trial design (C); indication (D); and, number of agents (E); sample size (number of trials) is shown below each box plot.

The median FI for trials with a binary outcome (*n* = 40) was 4.5 (range: 1–33; Table 2). Median FI appeared slightly higher for positive trials than reverse FI for negative trials [6 (1–33) vs. 3 (1–8)]. The median FQ was 0.026 (0.001–0.11), meaning that, on average, if the outcome for 2.6% of patients was reversed, that would be sufficient for a trial's primary comparison to gain (or lose) statistical significance. Positive trials tended to have a higher median FQ and larger range. Noninferiority trials tended to have lower FQ as compared to superiority trials for both positive and negative trials (Figure 2).

**TABLE 2:** Fragility indices of phase 3 pediatric cancer trials (*n* = 113).

	Binary response endpoint			Time‐to‐event endpoint		
	Positive trials FI	Negative trials FI	All trials FI	Positive trials SIFI**	Negative trials SIFI**	All trials SIFI**
Number of trials	17	23	40	24	49	73
Median FI or SIFI (range)	6 (1, 33)	3 (1, 8)	4.5 (1, 33)	11.5 (1, 56)	13 (0*, 61)	13 (0, 61)
Median FQ or SFQ (range)	0.05 (0.003, 0.11)	0.02 (0.001, 0.05)	0.026 (0.001, 0.11)	0.026 (0.005, 0.21)	0.029 (0, 0.13)	0.03 (0, 0.21)
Median number of patients LTFU (range)	N/A	N/A	N/A	5 (0, 21) [*n* = 7]	3 (0, 120) [*n* = 29]	3.5 (0, 120) [*n* = 36]

Abbreviations: FI, conventional fragility index; LTFU, lost to follow‐up; SIFI, survival‐inferred fragility index.

* This SIFI = 0 is from a trial that had a reconstructed *p*‐value of 0.0498. Because SIFI is an integer, the resulting SIFI value was 0.

** The absolute value of SIFI. This converts negative SIFI values to positive numbers.

### 3.3 Survival‐Inferred Fragility Index and Quotient for Trials Reporting a Time‐To‐Event Outcome

The median SIFI for trials with a time‐to‐event outcome (*n* = 73) was 13 (range: 0–61; Table 2 and Table S2). The median number of patients lost to follow‐up was 3 (0–120, *n* = 36 trials with known follow‐up). Nine (25%) of these 36 trials had a SIFI that was less than the number of patients lost to follow‐up. Among positive trials with a superiority design, there was a nonsignificant negative correlation between the absolute value of SIFI and reconstructed *p*‐value (*R* = −0.28, *p* = 0.23; Figure S2A) and a strong positive correlation between the absolute value of SIFI and sample size (*R* = 0.69, *p* = 0.0007; Figure S2B).

**FIGURE 3:**
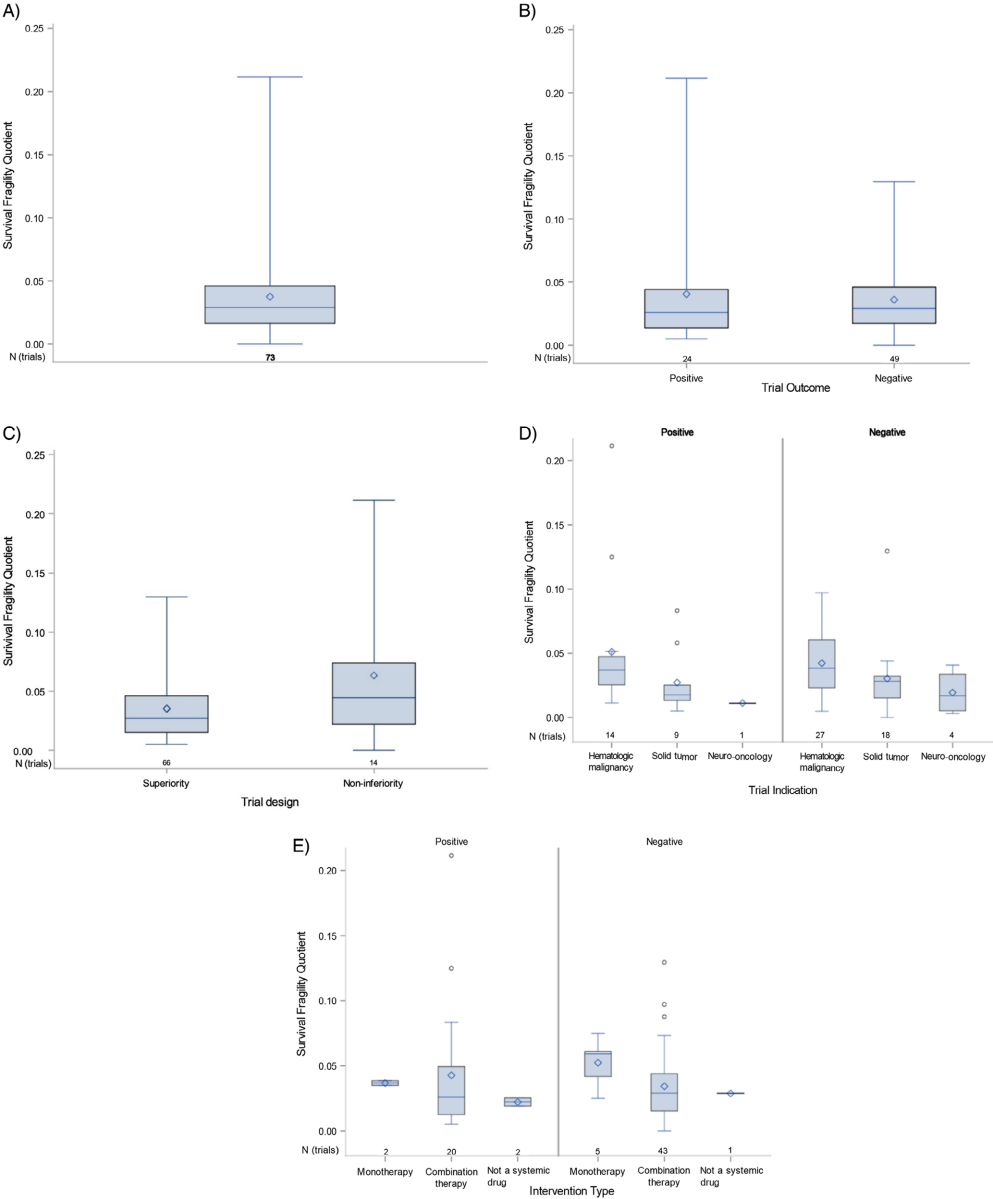
Box and whisker plots, with minimum, maximum, quartiles, and mean of survival fragility quotients for all trials: With time‐to‐event endpoints (A); according to trial outcome (B); trial design (C); indication (D); and, number of agents (E); sample size (number of trials) is shown below each box plot.

The median SFQ was 0.03 (0.0–0.21) (Table 2), meaning that, on average, if the outcome for 3% of patients were reversed, that would be sufficient for a trial to gain (or lose) statistical significance. There was a nonsignificant negative correlation between SFQ and trial sample size (*R* = −0.229, *p* = 0.051). SFQs by trial characteristic are shown in Figure 3. Hematologic malignancy trials had a larger sample size than solid tumor trials (mean: *n* = 500 vs. *n* = 307, respectively) and larger SIFI (15 vs. 9, respectively). However, the SFQs between trial indications are similar, that is, their relative fragility is similar (Figure 3D). Trials studying anticancer and supportive interventions had similar statistical fragility (median FQ/SFQ 0.03 (range: 0.01–0.13, *n* = 88) versus 0.04 (range: 0.01–0.09, *n* = 25)).

### 3.4 Trends in Fragility Parameters Over Time

**FIGURE 4:**
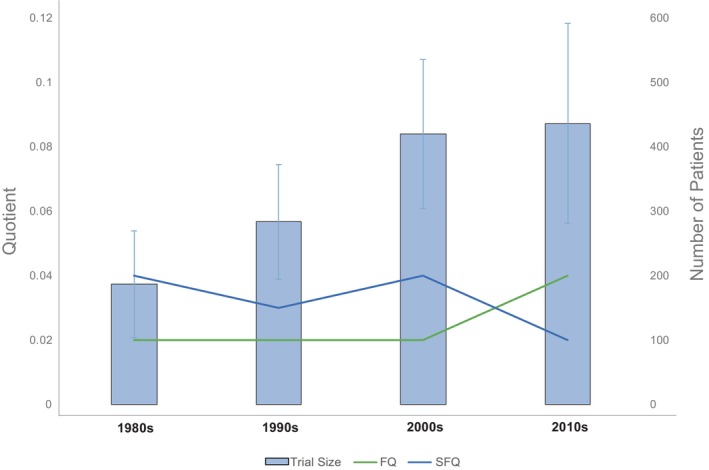
Mean and 90% confidence interval of trial sample size (left *y*‐axis; blue histogram), and fragility quotient (FQ) and survival fragility quotient (SFQ) (right *y*‐axis; green and blue lines, respectively), by decade (*n* = 9 for 1980s, *n* = 18 for 1990s, *n* = 35 for 2000s, *n* = 51 for 2010s).

The median FQ or SFQ for all trials did not appear to vary over our four decades of study despite increases in average trial size over time (Figure 4). With increasing trial size over time, median FI and SIFI increased by decade (Table S3). In this context, trial statistical significance appears contingent on outcomes for roughly the same proportion of patients regardless of the absolute number enrolled. Our subgroup analysis showed that trials with a time‐to‐event outcome appear to have lower SFQs over time (1980s: SFQ = 0.04; 2010s: SFQ = 0.02).

## 4 Discussion

This study is the first to comprehensively analyze the statistical fragility of phase 3 trials in pediatric oncology. Pediatric trials have unique statistical vulnerabilities which are often attributed to their smaller enrollment numbers when compared to adult studies. Here, we illustrate this issue is more complex and is not solely driven by patient numbers. Our pediatric oncology fragility indices were on par or less fragile compared with those reported in analyses of adult oncology trials [8, 12, 17] and other pediatric subspecialties [5, 18, 19]. There did not appear to be significant changes in trial fragility among subgroups or over time. Of note, there is no definitive threshold for a “significant” or “nonsignificant” FI or quotient. FI, SIFI, FQ, and SFQ require trial‐specific interpretation in the context of other trial metrics.

Trial size is often used as a proxy for statistical robustness. We did not find this to be true in our analysis. In our cohort of trials, we found that sample size did not significantly correlate with SFQ values, whereby smaller trials had similar or less statistical fragility as larger trials. For example, hematologic malignancy trials were on average 200 patients larger than solid tumor trials but had a similar SFQ. In addition, we found that FQs have not appreciably changed in the last four decades; the proportion of patients relative to trial size required to gain or lose statistical significance has remained relatively constant despite increases in average trial size over time. This finding is multifactorial, but it does strongly suggest that trials possess an innate degree of statistical fragility that has not been remedied by increasing study enrollment. It may be possible that investigators have shifted toward detecting smaller differences between groups over time, though this did not appear to be true based on our data.

For a given trial, the FI is often compared to the number of patients lost to follow‐up to lend a more complete picture of the robustness of the trial's result. As a rule of thumb, if the number of patients lost to follow‐up is greater than the absolute value of FI or SIFI, the trial results should be considered less robust. Several studies looking at cohorts of adult cancer trials have shown that approximately half or greater have a FI that is less than the number of patients lost to follow‐up [12, 20, 21]. This raises the concern that a trial may gain or lose statistical significance simply by decreasing study attrition. In our study, we found this to be true for only 25% of trials with reported data, which may represent a unique strength of pediatric trials. However, this estimate is potentially biased since follow‐up data were known in less than a third (36 of 113) of the trials. Investigators should continue to focus on retaining patients throughout the study period, which is likely a more effective way to improve statistical robustness than increasing trial enrollment.

Very few therapies have achieved FDA approval for a pediatric cancer indication in recent years. The differences in the regulatory landscape of adult and pediatric cancer can be in part attributed to rare disease prevalence, potential for more limited study data, low mutation burden with fewer actionable targets, high cost, and limited pharmaceutical incentives in pediatric cancer [22, 23]. Clinical decisions in pediatric cancer are more often based on data from early phase trials and fewer therapies advance to phase 3 testing. For example, Nader and colleagues found that only 2.6% of neuroblastoma trials over the last 10 years have been phase 3 trials [24]. Off‐label use to treat pediatric cancer has also become increasingly common [25]. The only trial in our cohort leading to an FDA approval studied dinutuximab in high‐risk neuroblastoma, which was approved based on the results from the phase 3 trial conducted by Yu and colleagues [26]. This trial had a SIFI of 3 and SFQ of 0.013. This trial was stopped early when a planned interim monitoring boundary for efficacy was crossed, which likely contributed to the high statistical fragility. Subsequently, the benefit of dinutuximab was validated with longer follow‐up and in a separate cohort [27, 28].

Several limitations apply to our study. In general, statistical fragility is a useful metric, but it only captures one aspect of trial “fragility”; other aspects not considered herein are effect size, adherence with treatment, and patient selection. Many trials identified in our PubMed search were unable to be included because they did not report sufficient information for analysis, limiting our sample size. Investigators are urged to consistently report all statistical and trial design parameters to allow for an objective interpretation by the reader. In addition, compared to more recent decades, there were a smaller number of analyzable trials from earlier decades, which may have introduced bias by giving more weight to recent trials. Fewer eligible trials from earlier decades may also reflect less robust reporting of phase 3 trial results before the implementation of consensus reporting guidelines, such as the CONSORT reporting guidelines [29]. Lastly, a small percentage of trials in our cohort were stopped early for success or futility, though the original level of significance from the trial design was used to calculate the FI/SIFI.

Designing and implementing statistically robust clinical trials is of the utmost importance to ensure that patients and their families have access to novel therapeutics in an efficient and safe way. Clinicians should challenge the notion that drug superiority can be determined solely by interpreting a *p*‐value; smaller trials should not be assumed to be inherently less statistically robust. We suggest that the FI and quotient be reported for all clinical trials to facilitate a nuanced data interpretation and aid in the translation of a dichotomous *p*‐value into a clinically meaningful number. Further work will be necessary to better understand achievable ways to increase trial statistical robustness in pediatric oncology and other rare diseases.

## Author Contributions

**Hannah E. Olsen:** conceptualization (equal), data curation (equal), formal analysis (equal), writing – original draft (equal), writing – review and editing (equal). **Pei‐Chi Kao:** conceptualization (equal), data curation (equal), formal analysis (equal), investigation (equal), methodology (equal), writing – review and editing (equal). **Caleb Richmond:** data curation (equal), investigation (equal), writing – review and editing (equal). **David S. Shulman:** conceptualization (equal), writing – review and editing (equal). **Wendy B. London:** conceptualization (equal), formal analysis (equal), investigation (equal), methodology (equal), writing – review and editing (equal). **Steven G. DuBois:** conceptualization (equal), formal analysis (equal), methodology (equal), resources (equal), writing – review and editing (equal).

## Conflicts of Interest

Steven G. DuBois reports travel expenses from Loxo Oncology, Roche, and Salarius, and consulting fees from Amgen, Bayer, and Jazz. Wendy B. London reports consulting fees from Merck Sharp & Dohme Corp, Jubilant Draximage Inc., and Y‐mAbs Therapeutics Inc. David S. Shulman reports consulting fees from Boehringer Ingelheim and Merlin Biotech.

## Supporting information


Data S1.

## Data Availability

The data that support the findings of this study are available from the corresponding author upon request.

## Appendix A List of Search Terms and Trial Criteria

The following search was inputted to PubMed to generate a list of potentially eligible trials: (phase 3 trial or phIII trial or randomized control trial or randomized trial or randomized) AND (pediatric oncology or pediatric cancer or pediatric malignancy or acute lymphoblastic leukemia or acute myeloid leukemia or sarcoma or osteosarcoma or Ewing sarcoma or neuroblastoma or Wilms tumor or rhabdomyosarcoma or retinoblastoma or Hodgkin's lymphoma or non‐Hodgkin's lymphoma, or lymphoblastic lymphoma or anaplastic large cell lymphoma or B‐cell lymphoma or diffuse large B‐cell lymphoma or Burkitt lymphoma or lymphoma or T‐cell lymphoma or CNS malignancy or astrocytoma or medulloblastoma or ependymoma or brainstem glioma or germ cell tumor or germinoma or seminoma or hepatoblastoma or glioma or rhabdoid tumor or atypical teratoid or rhabdoid tumor).

Filters were used for date (January 1, 1980—May 1, 2020), English language, and publication type (clinical trial). The age filter was not used and instead was manually assessed by review of title, abstract, and/or age reported in the trial text.
