# Supplementary material for: Statistical Fragility of Findings From Randomized Phase 3 Trials in Pediatric Oncology
Source: Cancer Med. 2024 Dec 15;13(24):e70356. doi: 10.1002/cam4.70356 (PMC11646933; doi:10.1002/cam4.70356)
Supplement: Supplementary file 1 — Data S1. [file CAM4-13-e70356-s001.docx]

**Supplemental Figure 1:** Example of applying a reclassification technique for a positive superiority trial (PMID 31454045) until statistical significance is lost. This published trial had 176 patients in the experimental group and 179 patients in the control group.

**Supplemental Figure 2:** Association between absolute value of SIFI and **A**) reconstructed p-value; and, **B**) trial sample size, for positive superiority trials with a time-to-event endpoint (n=20). Bubbles are shaded from yellow to red to reflect the increasing magnitude of the reconstructed hazard ratio (color scale shown along the right side of the figure).

**Supplemental Table 1:** Observed log rank test p-values and SIFI by trial design, R module (*Sifi* or *Neg_sifi*), and result.

**Supplemental Table 2:** Trial characteristics, SIFI, and patients lost to follow-up for 73 trials with a time to event outcome.

5-FU – 5-Fluorouracil; 6-MP – 6–mercaptopurine; ABVD – doxorubicin, bleomycin, vinblastine, dacarbazine; ADE – araC, daunorubicin, etoposide; ADCOMP: daunorubicin, asparaginase, methotrexate, cyclophosphamide, vincristine, prednisone; ADxE – cytarabine, liposomal daunorubicin, etoposide; AIE – cytarabine, idarubicin, etoposide; APO – doxorubicin, vincristine, prednisone, 6–mercaptopurine, and methotrexate; Ara–C – cytarabine; BCD – bleomycin, cyclophosphamide, dactinomycin; ASP – asparaginase; BuMel – busulfan, melphalan; CEM – carboplatin, etoposide, melphalan; CsA – cyclosporine; CTX – cyclophosphamide; CP – carboplatin; DDP – cisplatin ; DEX – dexamethasone; DI – Dose–intensified; DOX – Doxorubicin; ETOP – etoposide; EFRT – extended–field radiation therapy; EPI – epirubicin; EVVAIA – etoposide, vincristine, dactinomycin, ifosfamide, doxorubicin; GO – gemtuzumab ozogamicin; IE – ifosfamide and etoposide; HM – Hematologic malignancy; IB – low–dose cytosine arabinoside, 6–mercaptopurine, cyclophosphamide; ID – intermediate–dose; IL–2 – interleukin–2; IFN–α–2b – interferon alfa–2b; IT – intrathecal; IV – intravenous; LD – low dose; MAE – mitoxantrone, araC, etoposide; MAP – cisplatin, doxorubicin, methotrexate; MAPIE – cisplatin, doxorubicin, methotrexate, ifosfamide, etoposide; MAH – methotrexate, cytarabine, hydrocortisone; MOPP – mechlorethamine, vincristine, procarbazine, prednisone; MTX – methotrexate; NM – nonmetastatic disease; PBSC – peripheral blood stem cell; NO – Neuro–oncology; PRED – prednisone; RT – radiotherapy; SIFI – survival-inferred fragility index; SR – standard risk; ST – Solid Tumor; TEN – teniposide; TPCV – thioguanine, procarbazine, lomustine, vincristine; VAI – vincristine, dactinomycin, ifosfamide; VAIA – vincristine, dactinomycin, ifosfamide, doxorubicin; VACA – vincristine, dactinomycin, cyclophosphamide, doxorubicin; VAC – vincristine, dactinomycin, cyclophosphamide; VAC/VI – vincristine, dactinomycin, cyclophosphamide alternating with vincristine and irinotecan; VC – vincristine/carboplatin; VCE – vincristine/carboplatin/etoposide; WBC – white blood cell; WLI – whole lung irradiation

**Supplemental Table 3:** FI/FQ and SIFI/SFQ by decade.

FI – Conventional fragility index; SIFI – survival-inferred fragility index

FQ – Fragility quotient; SFQ – survival fragility quotient

**Supplemental Table 1**

|  | Trial result | |
| --- | --- | --- |
| Trial design | Positive | Negative |
| Superiority | (observed p<0.05)  *Sifi* (SIFI>0) | (observed p≥0.05)  *Neg_sifi* (SIFI<0) |
| Non-inferiority | (observed p≥0.05)*  *Neg_sifi* (SIFI<0) | (observed p<0.05)*  *Sifi* (SIFI>0) |

***** In the publications, investigators of the non-inferiority trials used H0: no difference in survival curves instead of using a H0: experimental is worse than standard by a pre-specified margin (a more widely accepted approach to testing non-inferiority).

**Supplemental Table 2**

| **PMID** | **Trial Result** | **Trial Design** | **Intervention** | **Control** | **Indication** | **Year of Publication** | **Number of Eligible Patients Randomized** | **SIFI** | **Absolute Value of SIFI** | **Number of Patients  Lost to Follow-up** |
| --- | --- | --- | --- | --- | --- | --- | --- | --- | --- | --- |
| 1403038 | Positive | Superiority | HDMTX/DOX/DDP | DOX/DDP | ST | 1992 | 198 | 1 | 1 |  |
| 2099751 | Positive | Superiority | High dose intermittent | Moderate dose continuous | ST | 1990 | 114 | 2 | 2 | 16 |
| 12697884 | Positive | Superiority | Chemotherapy and RT | RT Alone | NO | 2004 | 179 | 2 | 2 |  |
| 16129365 | Positive | Superiority | Megatherapy | Maintenance therapy | ST | 2005 | 295 | 2 | 2 |  |
| 20879881* | Positive | Superiority | Immunotherapy | Standard therapy | ST | 2010 | 226 | 3 | 3 |  |
| 9324339 | Positive | Superiority | CTX/VCR/DOX/MTX/Ara-C | CTX/HDMTX/VCR/PRED | HM | 1997 | 123 | 2 | 5 |  |
| 9440749 | Positive | Superiority | IVMTX/IVMP | LDMTX/IVMP | HM | 1998 | 699 | 8 | 8 |  |
| 23091096 | Positive | Superiority | Interval compressed | Standard | ST | 2012 | 568 | 8 | 8 |  |
| 31454045 | Positive | Superiority | Tandem transplant | Single transplant | ST | 2019 | 355 | 9 | 9 | 21 |
| 12594313 | Positive | Superiority | DOX/VCR/DDP/IE (NM) | DOX/VCR/DDP (NM) | ST | 2004 | 120 | 10 | 10 |  |
| 28259608 | Positive | Superiority | BuMel | CEM | ST | 2017 | 598 | 13 | 13 | 2 |
| 8652810 | Positive | Superiority | Intensive timing | Standard timing | HM | 1996 | 589 | 15 | 15 | 2 |
| 10086723 | Positive | Superiority | HDASP | Control | HM | 1999 | 484 | 15 | 15 |  |
| 9614257 | Positive | Superiority | Augmented therapy | Standard therapy | HM | 1998 | 311 | 16 | 16 |  |
| 15744348* | Positive | Superiority | IV 6-MP | No IV 6-MP | HM | 2005 | 877 | 23 | 23 |  |
| 11929760 | Positive | Superiority | Erwinia-asparaginase | E coli-asparaginase | HM | 2002 | 700 | 27 | 27 |  |
| 32492302* | Positive | Superiority | Rituximab + chemotherapy | Chemotherapy | HM | 2020 | 328 | 41 | 41 |  |
| 26888258 | Positive | Superiority | DEX | PRED | HM | 2016 | 3720 | 45 | 45 |  |
| 15952999* | Positive | Superiority | DEX | PRED | HM | 2005 | 1603 | 56 | 56 | 5 |
| 23575054 | Positive | Superiority | Augmented therapy | Standard therapy | HM | 2012 | 317 | 15 | 15 |  |
| 18802150 | Positive | Non-inferiority | SR-VACA | SR-VAIA | ST | 2008 | 155 | -9 | 9 | 0 |
| 21480469 | Positive | Non-inferiority | 12 Gy | 18 Gy | HM | 2011 | 471 | -9 | 9 |  |
| 2642543 | Positive | Non-inferiority | 6 Cycles | 18 Cycles | HM | 1989 | 104 | -22 | 22 |  |
| 18368070 | Positive | Non-inferiority | ALL-MB 91 protocol | ALL-BFM 90m protocol | HM | 2008 | 713 | -29 | 29 | 8 |
| 24335695 | Negative | Superiority | Dose-intensive chemotherapy | Standard chemotherapy | NO | 2014 | 328 | -1 | 1 | 9 |
| 28384657 | Negative | Superiority | Metronomic chemotherapy | Placebo | ST | 2017 | 108 | -1 | 1 | 0 |
| 2910421 | Negative | Superiority | IT MAH | CTX/ASP post-induction | HM | 1989 | 413 | -2 | 2 | 30 |
| 22665535 | Negative | Superiority | TPCV | CP/VCR | NO | 2012 | 274 | -2 | 2 | 3 |
| 10894865 | Negative | Superiority | CDDP/DOX | CDDP/5-FU/VCR | ST | 2000 | 173 | -3 | 3 |  |
| 11455971 | Negative | Superiority | 12 cycles over 30 weeks | 12 cycles over 24 weeks | HM | 2001 | 490 | -3 | 3 | 1 |
| 7751881 | Negative | Superiority | ADCOMP | LSA_2_L_2_ | HM | 1995 | 281 | -4 | 4 |  |
| 9508171 | Negative | Superiority | MOPP/ABVD | ABVD+EFRT | HM | 1998 | 111 | -4 | 4 |  |
| 18308250 | Negative | Superiority | Rapid | Standard | ST | 2008 | 262 | -4 | 4 | 21 |
| 18802150 | Negative | Superiority | HR-EVAIA | HR-VAIA | ST | 2009 | 492 | -4 | 4 | 0 |
| 27324280 | Negative | Superiority | Zoledronate | Control | ST | 2016 | 315 | -4 | 4 | 3 |
| 8427957 | Negative | Superiority | TEN/Ara-C | PRED/DOX | HM | 1993 | 99 | -5 | 5 |  |
| 31553693* | Negative | Superiority | BuMel | VAI-WLI | ST | 2019 | 287 | -6 | 6 |  |
| 7799011 | Negative | Superiority | 8 in 1 regimen | Control | NO | 1995 | 172 | -7 | 7 |  |
| 3539306 | Negative | Superiority | Bactrim | No Bactrim | HM | 1987 | 120 | -8 | 8 | 1 |
| 20212252 | Negative | Superiority | Intermittent 6-MP/MTX | Continuous 6-MP/MTX | HM | 2010 | 544 | -8 | 8 | 2 |
| 31283407 | Negative | Superiority | ADE+MAE | IB | HM | 2019 | 330 | -8 | 8 | 4 |
| 19450759 | Negative | Superiority | Early CsA | Conventional CsA | HM | 2009 | 152 | -9 | 9 |  |
| 22730539 | Negative | Superiority | GO | No GO | HM | 2012 | 120 | -9 | 9 | 0 |
| 2924255 | Negative | Superiority | WBC 1500-3000 | WBC 3000-4500 | HM | 1989 | 434 | -10 | 10 | 42 |
| 8448756 | Negative | Superiority | VAC/DOX + radiation | VAC + radiation | ST | 1993 | 310 | -10 | 10 | 2 |
| 8614385 | Negative | Superiority | IV MTX | Oral MTX | HM | 1996 | 164 | -10 | 10 | 5 |
| 23704089 | Negative | Superiority | ADxE | AIE | HM | 2013 | 521 | -13 | 13 | 10 |
| 28649001* | Negative | Superiority | VCE | VC | NO | 2017 | 490 | -13 | 13 | 6 |
| 30091945 | Negative | Superiority | VAC/VI | VAC | ST | 2018 | 448 | -16 | 13 |  |
| 6202851 | Negative | Superiority | DDP | BCD | ST | 1984 | 108 | -14 | 14 |  |
| 17658395* | Negative | Superiority | Delayed Intensification | Standard therapy | HM | 2007 | 191 | -14 | 14 | 4 |
| 23890779 | Negative | Superiority | Purged PBSC | Non-purged PBSC | ST | 2013 | 486 | -14 | 14 | 0 |
| 29941280* | Negative | Superiority | VAI/DOX | VAI | ST | 2018 | 484 | -14 | 14 |  |
| 16170158 | Negative | Superiority | DEX | PRED | HM | 2005 | 359 | -15 | 15 |  |
| 16983120 | Negative | Superiority | 2 consolidation cycles | Biphasic 6 week consolidation | HM | 2006 | 390 | -15 | 15 | 2 |
| 17227995 | Negative | Superiority | Dose-intensified therapy | Conventional therapy | ST | 2007 | 497 | -16 | 16 |  |
| 30442501 | Negative | Superiority | Dinutuximab+IL-2 | Dinutuximab | ST | 2018 | 406 | -16 | 16 |  |
| 15659500* | Negative | Superiority | IDMTX/HiDAC | APO | HM | 2005 | 175 | -17 | 17 |  |
| 27569442 | Negative | Superiority | MAPIE | MAP | ST | 2016 | 618 | -17 | 17 | 3 |
| 30545921* | Negative | Superiority | CTX/ETOP | CTX/Ara-C/6-MP | HM | 2019 | 732 | -18 | 18 |  |
| 20679620 | Negative | Superiority | Vinblastine | No vinblastine | HM | 2010 | 217 | -19 | 19 | 2 |
| 26033801 | Negative | Superiority | MAP+IFN-α-2b | MAP | ST | 2015 | 716 | -19 | 19 | 40 |
| 2064956 | Negative | Superiority | Daunorubicin | No daunorubicin | HM | 1991 | 630 | -20 | 20 | 1 |
| 22665534 | Negative | Superiority | VAI+CP/ETOP/EPI | VAI | ST | 2012 | 457 | -20 | 20 | 12 |
| 19349548 | Negative | Superiority | DI-VDC/IE | Standard VDC/IE | ST | 2009 | 478 | -21 | 21 |  |
| 9704737 | Negative | Superiority | IDM + Ara-C | IDM | HM | 1998 | 427 | -23 | 23 |  |
| 14675406 | Negative | Superiority | HDMTX + IT MTX | IT MTX | HM | 2004 | 1513 | -25 | 25 |  |
| 11230471 | Negative | Superiority | HDASP | SDASP | HM | 2001 | 610 | -28 | 28 | 7 |
| 12843002 | Negative | Superiority | Thioguanine | Mercaptopurine | HM | 2003 | 474 | -28 | 28 | 1 |
| 24727815 | Negative | Superiority | DEX | PRED | HM | 2014 | 1941 | -61 | 61 | 120 |
| 2448428* | Negative | Non-inferiority | BCD | DOX/DDP | ST | 1988 | 120 | 0 | 0 |  |
| 29148893 | Negative | Non-inferiority | Reduced dose intensity | Standard dose intensity | HM | 2018 | 1163 | 21 | 21 |  |
| 15486066* | Negative | Non-inferiority | 4 hour MTX infusion | 24 hour MTX infusion | HM | 2005 | 364 | 22 | 22 | 14 |

*Trial was stopped early for success or futility.

**Supplemental Table 3**

|  | **SIFI n** | **SIFI (trials with time-to-event endpoint, n=73),**  **median (range)** | **FI**  **n** | **FI (trials with binary endpoint, n=40),**  **median (range)** | **SFQ**  **n** | **SFQ (trials with time-to-event endpoint, n=73),**  **median (range)** | **FQ**  **n** | **FQ (trials with binary endpoint, n=40),**  **median (range)** | **All trials**  **n** | **FQ (all trials, n=113),**  **median (range)** |
| --- | --- | --- | --- | --- | --- | --- | --- | --- | --- | --- |
| **Decade of Publication**  **1980-89**  **1990-99**  **2000-09**  **2010-20** | 6  15  23  29 | 9 (0, 22)  8 (1, 23)  15 (2, 56)  13 (1, 61) | 3  3  12  22 | 3 (1, 5)  3 (2, 5)  4 (2, 11)  5 (1, 33) | 6  15  23  29 | 0.04 (0, 0.21)  0.03 (0.005, 0.06)  0.04 (0.006, 0.097)  0.02 (0.003, 0.13) | 3  3  12  22 | 0.02 (0.009, 0.05)  0.02 (0.016, 0.04)  0.02 (0.006, 0.07)  0.04 (0.001, 0.11) | 9  18  35  51 | 0.02 (0, 0.21)  0.03 (0.005, 0.06)  0.03 (0.006, 0.097)  0.03 (0.001, 0.13) |

**Supplemental Figure 1 Supplemental Figure 2**
